# Intelligence

**Published:** 1830-06

**Authors:** 


### INTELLIGENCE.

### COLLEGE OF PHYSICIANS, *May* 3.

THE chair this evening was taken by Dr. ROBERTS, when a paper was read by the registrar, Dr. HAWKINS, consisting of

*Observations on the Blood; by* WILLIAM STEVENS, M.D.

The author remarked that there is often, in the West Indies, a malignant form of yellow fever, in which it is obvious, from the symptoms during life, as well as from the appearance presented after death, that the disease has had its chief seat in the fluids. The cause of death, he thinks, under such circumstances, only becomes apparent when we open the heart and observe its contents. In this we find " a dissolved fluid," in place of blood, which is black as ink, and unfit for the purposes of life. These and various other circumstances induced Dr. Stevens to pay particular attention to the blood, and to make a number of experiments upon it, the results of which we subjoin.

The importance of the blood, and of the changes which it presents, have been comparatively neglected for nearly a century; and to this the author attributes the assumed fact of our " going back" in the theory of fever, at a time when such brilliant discoveries have been making in other departments of medical science. On examining the blood of those who had died of yellow fever, the following changes presented themselves.

1. It was more fluid than natural; a circumstance partly attributed to an excess of serum, and partly to the fibrin not being found in its usual quantity. Besides this, the colouring matter was observed to be frequently detached from the globules, and dissolved in the serum; nor can this colouring matter be separated from the serum by filtration, or any other mechanical means. As the disease advances, however, the red colour is lost, and the whole circulating current becomes black, as well as thin.

2. The whole mass of blood, both in the arteries and veins, was changed to this black colour. Dr. S. has often taken black vomit from the stomach, and blood from the heart, and these have resembled each other so much as to render it almost impossible to distinguish them.

3. In bad fevers the saline matter appears to be exhausted faster than it enters the circulation; the blood losing its saline taste; of which circumstance the black colour is found to be a certain proof.

4. The blood, though dissolved, is not putrid; but dissolution is regarded by the author as the first step in the putrefactive process. Such dissolved state is held to be the cause, and not the consequence, of death; being sometimes present during life. But it is stated to be the effect, not the cause, of fever. Now, as this dissolved condition is regarded by Dr. Stevens as being frequently the sole cause of the fatal termination, so it became an object with him to discover some agent capable of preventing this change.

Saline matters, it is observed, are generally antiseptic; during fever they are diminished in quantity; and hence it was thought that their administration, in the form of medicine, might be advantageous. Accordingly this was done; and, after repeated trials, the author became convinced that, when properly administered, they have "a specific effect" in preventing the dissolution of the blood.

Witnessing these results, and recollecting that various neutral salts pass into the circulation unchanged, Dr. Stevens was led to try what effect would be produced when these and other substances were mixed with the blood while it was yet warm and fluid.* The results were, 1st, that acids, as a general rule, rendered the blood darker; and this in proportion to their strength. When any of the strong acids was mixed with a little water, and added to recently drawn blood, this immediately became changed from red to black. Even the vegetable acids produce this effect.

2dly. The alkalies produce a similar change, though not in so remarkable a degree.

3dly. The neutral salts immediately give to venous blood a bright scarlet colour. This effect likewise resulting although the alkali might be a little in excess, as in the subcarbonate of soda.

4thly. Even the black and morbidly attenuated blood taken from the heart in fatal cases of yellow fever, was similarly changed into a bright red fluid by the addition of neutral salts.

Dr. Stevens proceeded to state, that he intended to enter on the subject more fully in a work which he is about to publish; when he will endeavour to prove that black is the natural hue of the colouring principle of the blood, and that the various properties resident in blood depend on the quantity and condition of its saline impregnation.

In drawing his inferences, the Doctor argued that, in violent fevers, even when proper means are used, chemical changes nevertheless frequently take place in the circulating fluids, and that these changes "are almost always the sole cause of the mortality." The deterioration of the blood, in the fevers of hot climates, is said to be very obvious, and to occur in the milder forms of the disease witnessed in this country, as proved by the experiments of Dr. R. Clanny, of Sunderland.

As the counterpart of this, it is held that, when efficient means are used "to protect the organs" during the early stage, and proper diet and saline medicines are subsequently exhibited, the bad symptoms are generally warded off. These are supposed to act chiefly by preventing the "dissolution" of the blood till the fever abates and the danger is gone. A method of treatment, founded on these views, is stated to have been remarkably successful, both in the hands

* The best mode of conducting this experiment is to mix the agent so employed with a little water, and add to it the blood before it coagulates.

of the author and others. Thus, fever prevailed at Trinidad in 1828: the patients were bled and purged freely at the commencement; then they had neutral salts; during their convalescence, quina; and one of Dr. Stevens's correspondents, Mr. Greatrex, stated that, of 340 cases so treated not one proved fatal. On the contrary, emetics, calomel, antimony, opium, and acids, are represented as increasing the evils they are intended to relieve; and, in fact, as greatly augmenting the rate of mortality in the fevers of hot climates. The Rochelle salt, and the carbonate of soda, were particularly mentioned by Dr. Stevens, though not to the exclusion of "other active saline medicines;" and, in conclusion, he stated that, since the treatment above alluded to had been adopted, the yellow fever had been in a great measure disarmed of its terrors.

---

### COLLEGE OF SURGEONS.

Mr. Guthrie, in completing the surgical part of his Lectures, as Professor of Anatomy and Surgery to the College, on Saturday last, showed the instrument used for breaking up stone in the bladder, with an improvement which he said he considered of the greatest importance, as far as regarded the safety of the patient. It consisted in a peculiar manner of fixing the shaft of the forceps, by means of which the instrument could be taken to pieces, either when closed or open in the bladder, and each piece of which it was composed might be withdrawn separately. He said he was led to make this improvement in consequence of the instrument having been clogged when used by him near five years ago, and he was the first surgeon who had used it in this country. He had been informed that two persons on the continent had lost their lives in consequence of the instrument having, by some accident, become fixed when open in the bladder, so that it could not be withdrawn. These accidents could now be prevented; and the principal, indeed only, objection to the use of the instrument, in his mind, was removed. It was to the ingenuity of Mr. Weiss that he was indebted for putting his suggestion into execution.

---

### LONDON UNIVERSITY.

*Distribution of Prizes. President*, Sir James Graham.

This gratifying ceremony took place on the 15th, in the presence of numerous spectators. The following are the names of the students who were successful candidates.

Professor Thomson's Class (*Materia Medica and Therapeutics*).

Gold Medal.—Mr. Pidwell.
First Silver Medal.—Mr. Hulme Edge.
Second Silver Medal.—Mr. Cheetham.

In Professor Conolly's (*Nature and Treatment of Diseases*).

Gold Medal.—Mr. F. R. Taylor, of Tortola.
First Silver Medal —Mr. H. O. Stephens, of Bristol.
Second Silver Medal.—Mr. R. Garner, of Staffordshire.

In Professor Pattison's (*Anatomy*).

Gold Medal.—Mr. E. Merrion, of Rye, Sussex.
First Silver Medal.—Mr. H. Cooper, of Tranby, near Hull.
Second Silver Medal.—Mr. T. Bidwell, of Penzance.

In Professor Bell's (*Physiology*).

Gold Medal.—Mr. W. Evans, of Dryclough, Lancashire.
First Silver Medal.—Mr. H. Cooper, of Tranby, near Hull.
Second Silver Medal.—Mr. T. G. Wright, of Stockton-upon-Tees.

In Professor Bell's (*Surgery*).

Gold Medal.—Mr. R. Garner, of Staffordshire.
First Silver Medal.—Mr. W. Evans, of Dryclough, Lancashire.
Second Silver Medal.—Mr. H. Cooper, of Tranby, near Hull.

In Professor Davis's (*Midwifery*).

Gold Medal.—Mr. F. R. Taylor, of Tortola.
First Silver Medal.—Mr. T. Wakefield, of Judd Place.
Second Silver Medal.—Mr. T. G. Wright, of Stockton-upon-Tees.

In Professor Turner's (*Chemistry*).

Gold Medal.—Mr. J. N. Huddleston, George street, Euston square.
First Silver Medal.—Mr. J. P. Wall, of Hertfordshire.
Second Silver Medal.—Mr. W. Cooks, of Warwick.

In Professor Grant's (*Comparative Anatomy*).

Gold Medal.—Mr. N. Esdell, of Twyford.
First Silver Medal.—Mr. E. Blackmore, of Manchester.
Second Silver Medal.—Mr. Benjamin Phillips.

In the Class of J. R. Bennett, Esq. (*Practical Anatomy*).

Gold Medal.—Mr. E. J. Queckett, of Langport, Somersetshire.
First Silver Medal.—Mr. C. Nelson, of Lees.
Second Silver Medal.—Mr. Blackmore, of Manchester.

Many of the students obtained certificates of honour, to whom medals were not awarded. Mr. Richards and Mr. Garner were precluded from receiving prizes upon this occasion, as they had last year received the medals in the same classes in which they now again distinguished themselves.

We select the following answers, given by Mr. Pidwell to some of the questions proposed by Professor Thomson:

*Question* 4. What are the effects of Strychnia on the animal economy? on what set of nerves does it chiefly operate? in what diseases is it indicated? State the combination for securing its influence, the dose at first, and to what extent it may be carried.

*Answer*. Strychnia produces in the animal economy tetanic convulsions, or violent contractions of the spinal muscles and the extensors of the lower extremities, by which the body is bent backwards, and the limbs rendered rigid. The convulsions cease for a short time, and then commence again, the breathing is affected, and the animal dies. On opening the body, the right side of the heart is found gorged with black blood. Strychnia acts on the motor nerves, particularly on the spinal nerves. It is indicated in paraplegia arising from the effect of carbonate of lead; or indeed it may be given in paralysis of that part arising from any other cause, provided there is not much inflammation existing at the time. It has been said that strychnia operates more particularly on those muscles which are diseased in their action. The sulphate is a good form of the medicine. Dose at first not more than one fourth of a grain, but it may be carried to the extent of two or three grains.

Q. 5. When strychnia kills, what post-mortem appearances enable you to assert that it was the cause of death? What is the supposed antidote of strychnia?

*A.* When strychnia kills, the appearance of the lungs and the empty state of the arteries indicate the agent. Should the igasauric acid have been combined with it, as in nux vomica, this may be tested by means of the sulphate of copper. The best antidote of strychnia is iodine, which renders it inert.

*Q.* 16. A man who has been suffering under depression of spirits dies suddenly: a small phial is found near the body, completely emptied of liquid, but having an odour that induces you to suspect that he has poisoned himself with Hydrocyanic acid. Describe this odour, the appearances of the body, the manner in which the contents of the stomach and the apparently empty phial should be tested, to demonstrate the truth of your suspicion.

*A.* In the first place, I should take a cork cut at the end, so that it should be perfectly fresh; then moisten it with a solution of potassa and proto-sulphate of iron; insert the cork thus moistened into the mouth of the phial; apply the hand to the phial for a few minutes, and then, taking out the cork, touch the surface that had been moistened with the potassa and sulphate of iron, and next with sulphuric acid: if hydrocyanic acid were present in the bottle, the end of the cork will present the colour of Prussian blue. The odour in the phial, and in the body of the patient when opened, would be, in case the poison were prussic acid, like the smell of laurel leaves, or the kernels of the peach, or the young tops of the black thorn when bruised: it is said to resemble that of the peach blossom, but this is not exactly so, as the oil of almonds has that smell more particularly. The most remarkable appearance about the body, if the person has not been dead long, is the apparent life of the eye and its brilliancy. On dissection, the heart is found gorged with blood of a dark colour. The contents of the stomach should be collected, and treated with distilled water acidulated, and then filtered. To the filtered fluid must be added, first a little potassa, then the solution of proto-sulphate of iron, and last sulphuric acid, on the addition of which you will see the Prussian blue make its appearance, if hydrocyanic acid was present.

*Q.* 22. When a person is poisoned by bichloride of mercury, what do you expect to see in the heart?

*A.* I should expect to find the valves of the aorta inflamed.

It is the intention of the Council to grant medical diplomas in the University. Upon this subject the following document has been issued:

"*Medical Diploma.* In the 'Second Statement by the Council,' published before the opening of the University in 1828, it is announced that, 'besides certificates of the professors, the University will grant certificates of general proficiency in literature and science. Every student will be required to produce a certain number of Professors' certificates, before he can be allowed to enter upon the examination for the general certificate.' The object in granting this certificate, is to put the student in possession of a document which shall be an evidence of his having acquired at the University a certain amount of knowledge in the different departments of general and professional education. But, to make the document practically useful, it must have such a designation as the person obtaining it can conveniently affix to his name and be called by; as is the case when he takes a degree at an incorporated university. The Council have been for some time engaged in considering the qualifications, conditions, and the form under which this diploma shall be granted in the different departments of education; they have not yet settled what these shall be in any other than the medical school, but expect to be able to announce the whole scheme before the conclusion of the present session. Having come to a decision upon the medical diploma, the Council have thought it advisable to make it known, before the medical students of the present session shall be dispersed. It has been resolved that the general university certificate shall be granted in the medical school under the following conditions and regulations, and that it shall be called 'The Diploma of Master of Medicine and Surgery in the University of London,' (which may be thus translated and abbreviated, M. Med. et Chir. U. L.)

"1. That the candidate shall be twenty-one years of age. 2. That he shall have attended lectures on professional subjects, during three academical sessions of this University; or two sessions at this University, and one winter session of at least five months' duration in any established school at home or abroad. 3. That he shall have acquired certificates of honour in the following classes in the University: Practice of Medicine; Anatomy; Physiology; Surgery; Midwifery, and Diseases of Women and Children; Materia Medica; Botany; Chemistry; and Anatomical Demonstrations and Dissections. 4. That, in the year which may be spent out of the University, he shall have attended lectures on two different professional subjects, each course of such lectures being of at least five months' duration. 5. That he shall have attended the medical practice of an hospital, containing at least 100 beds, for twelve months; and the surgical practice of the same hospital, or another hospital of the same number of beds, for the like period of twelve months. 6. That he shall be required to translate a passage in writing from Celsus, Gregory, Heberden, or other Latin medical author. 7. That, having complied with the preceding regulations, he shall write an essay in the English language on some professional subject, chosen by himself, and approved of by the faculty of medicine before the essay is composed. That this essay shall be read in whole or in part, as the faculty may desire, at a public meeting in the University; and that the candidate shall be called upon to explain or defend the doctrines maintained in his essay. That he shall also make an anatomical demonstration, and be examined upon any part of his professional studies on which the faculty of medicine may think proper to propose questions.

"In proposing the above regulations for conferring an honorary distinction, suited to surgeons and general practitioners, the Council have thought it proper to require attendance on those classes only which are necessary for obtaining the diploma of the College of Surgeons of London, and Society of Apothecaries. But they are desirous that the attention of medical students should also be particularly directed to the subjects of clinical medicine, comparative anatomy, and medical jurisprudence: and it will also be a great recommendation to candidates that they should possess some knowledge of mathematics, natural philosophy, and natural history. The scheme of instruction in the University affords ample opportunity for such studies; and a diligent pupil, during the period prescribed by the regulations, may obtain respectable knowledge in several of those departments, without interfering with the more direct object of his pursuits. The diploma will be conferred in public on the 23d of December in each year, or the 22d, if the 23d be a

Sunday; and the examinations will commence on the corresponding day of the preceding week. The essay must be sent to the secretary of the Faculty of Medicine on or before the 15th of November, and must be signed by the candidate, with a declaration that it is wholly his own composition. In the event of students leaving England, or in other cases of emergency, the diploma will be granted at other periods of the session, if the candidates possess the necessary qualifications. In selecting the designation, care has been taken to avoid all interference with the titles and privileges conferred by chartered bodies. The value of the diploma to the possessor of it will depend upon its being known to be granted to those only who, after a strict examination, prove themselves worthy of such a distinction.

"The following extract from a report of the Faculty of Medicine contains their views as to the good effects upon the education of medical students which this measure may tend to produce: 'The medical profession is divided into three classes: viz. physicians, surgeons, and general practitioners. The latter form by far the greater body; and until this University can give a physician's degree, not many of those destined for that branch of the profession can be expected to take any considerable part of their education in its medical school. Under the appellation of general practitioners are included two distinct classes of medical men. One of these consists of practitioners who hold a highly respectable rank in the profession, and who have devoted much time, labour, and money to their professional education; men possessed of some attainments in the collateral sciences, and who, practising their profession in a liberal and scientific spirit, have the highest claim to the confidence of the public. Another class bearing the same appellation, consists of those who have acquired the right to practise by possessing only the *minimum* of knowledge by which the licence can be obtained, earned by the smallest possible expenditure of time and labour, and who consequently have very imperfect professional attainments. The public possess so little knowledge of the details of a medical and surgical education, that all the most serious duties of the profession are commonly confided, without inquiry, to any one who calls himself a general practitioner, and to such hands, especially in the country, the largest portion of professional duty and responsibility is intrusted. It becomes, therefore, a great duty for the University to endeavour to remedy the evil, as far as it has the means of doing so, by holding out this honorary distinction as encouragement to general practitioners to follow such a more extended course of study as the science they profess, and as the public interest require."

"(By order of the Council,)

"LEONARD HORNER, *Warden.*

"*University of London; April* 24, 1830."

---

Mr. Jewel has been unanimously elected one of the accoucheurs to the St. George's and St. James's Dispensary.

---

*To the Editor of the London Medical and Physical Journal.*

*Reigate; May* 11*th*, 1830.

Sir: An association of medical men in Surrey, under the title of "The Surrey Benevolent Medical Society," and meeting yesterday at Epsom, being desirous of doing some small justice to a worthy, an able, and an honourable man, agreed to address a letter to Dr. BURROWS, of which the following is a copy; and at the same time directed me to request the favor of you to insert it in the next Number of your Journal.

I have the honour to be, Sir, your most obedient servant,

THOMAS MARTIN, *Secretary*.

*To Dr. Burrows.*

*Epsom; May 10th*, 1830.

Sir: The members of the Surrey Medical Society, in consequence of circumstances which have occurred, and from which you have been most strangely and unjustly misrepresented and traduced in the public newspapers, beg permission to express their sympathy and the assurance of their most cordial and undiminished regard for you, as individually their friend, the friend of our society, of medical science, and of humanity.

Conscious of your integrity, and of the blameless as well as praiseworthy tenor of your professional conduct upon all occasions, you hardly stand in need of this assurance from such humble individuals; but you have been so hardly and unjustly treated by the public press in particular, that they consider your professional brethren may, without impropriety, state to you that they are not forgetful of your former labours in behalf of the general practitioner; of the merits of your admirable writings; and of the friendship and kindness they have collectively and individually experienced at your hands.

Wishing you a long career of happiness, success, and of meritorious exertion in behalf of your suffering fellow-creatures, they remain your faithful friends and servants,

John Parrott, Thomas B. Toovey,
Thomas Martin, John Winslow Mayd,
John N. Shelley, J. H. Montagu,
George Fletcher, Wm. Heart,
James Tunstall, Wm. Chaldecott,
Geo. Bottomley, Thomas Steele,
Joseph Ward, Thomas Smith,
Edward Wallace, Alfred Hardwick.
Charles Cooper,

### MONTHLY LIST OF MEDICAL BOOKS.

### METEOROLOGICAL JOURNAL,

By Messrs. HARRIS and Co. Mathematical Instrument Makers, 50, High Holborn.

| April | Rain gauge. | Moon. | Thermom. | | | Barometer. | | De Luc's Hygrom. | | Winds. | | Atmospheric Variations. | | |
|---|---|---|---|---|---|---|---|---|---|---|---|---|---|---|
| | | | 9 a.m. | max. | min. | 9 A.M. | 10P.M. | 9A.M. | 10P.M. | 9 A.M. | 10P.M. | 9 a.m. | 2 p.m. | 10 p.m. |
| 20 | .24 | | 52 | 57 | 46 | 29.58 | 29.81 | 59 | 57 | W | NW | Rain | Fine | Fine |
| 21 | .02 | | 57 | 58 | 51 | .76 | .50 | 57 | 59 | W | SW | Rain | Fine | Show'ry |
| 22 | | ● | 56 | 58 | 52 | .43 | .29 | 59 | 66 | WSW | WSW | Cloudy | Rain | Show'ry |
| 23 | .33 | | 55 | 55 | 49 | .20 | .14 | 69 | 65 | SW | SW | Rain | Rain | Cloudy |
| 24 | .15 | | 55 | 57 | 46 | .12 | .52 | 63 | 55 | W | W | Rain | Fine | Fine |
| 25 | | | 51 | 60 | 43 | .76 | .94 | 55 | 52 | W | NW | Fine | Fine | — |
| 26 | | | 56 | 62 | 45 | 30.06 | 30.07 | 50 | 48 | W | WSW | — | — | — |
| 27 | | | 59 | 65 | 48 | .09 | .03 | 45 | 46 | SW | ESE | — | — | — |
| 28 | | | 62 | 65 | 52 | 29.97 | 29.91 | 46 | 47 | ESE | ESE | — | — | — |
| 29 | | ☽ | 62 | 68 | 54 | .86 | .80 | 47 | 48 | ESE | SE | — | — | — |
| 30 | | | 67 | 72 | 55 | .73 | .79 | 48 | 48 | SE | WSW | — | — | — |
| May | | | | | | | | | | | | | | |
| 1 | | | 59 | 66 | 46 | .83 | .88 | 47 | 45 | WSW | WSW | — | — | — |
| 2 | | | 57 | 63 | 47 | .93 | 30.00 | 45 | 45 | W | W | — | — | — |
| 3 | | | 56 | 60 | 47 | 30.05 | .02 | 45 | 45 | W | WSW | — | — | — |
| 4 | | | 54 | 63 | 48 | .02 | 29.98 | 45 | 46 | SW | SE | — | — | — |
| 5 | | | 62 | 71 | 56 | 29.94 | .91 | 46 | 46 | SE | SE | — | — | — |
| 6 | | | 70 | 77 | 60 | .74 | .63 | 46 | 46 | SSE | ESE | — | — | — |
| 7 | | ○ | 70 | 75 | 55 | .47 | .48 | 46 | 48 | ESE | SSW | — | — | — |
| 8 | | | 58 | 59 | 47 | .43 | .30 | 48 | 50 | ESE | SE | — | Rain | Cloudy |
| 9 | | | 50 | 54 | 48 | .30 | .22 | 53 | 55 | NW | NW | Rain | Rain | Rain |
| 10 | | | 53 | 55 | 44 | .25 | .47 | 55 | 55 | W | NE | Cloudy | Rain | Cloudy |
| 11 | .50 | | 50 | 54 | 45 | .56 | .61 | 55 | 54 | NNE | N | Cloudy | Cloudy | Clou y |
| 12 | | | 52 | 56 | 46 | .64 | .72 | 54 | 55 | N | NE | Fine | Fine | Cloudy |
| 13 | | | 50 | 54 | 44 | .81 | .94 | 55 | 54 | N | NNE | Cloudy | Cloudy | Cloudy |
| 14 | | | 52 | 57 | 48 | 30.02 | 30.05 | 54 | 54 | NE | ENE | Cloudy | Fine | Fine |
| 15 | | ☾ | 54 | 63 | 44 | .05 | .09 | 54 | 50 | SSE | SSW | Fine | Fine | Cloudy |
| 16 | | | 57 | 68 | 48 | .09 | .09 | 50 | 50 | W | W | — | — | — |
| 17 | | | 60 | 73 | 58 | .07 | 29.98 | 50 | 48 | SW | WSW | — | — | — |
| 18 | | | 70 | 72 | 55 | 29.86 | .75 | 46 | 45 | WSW | WSW | — | — | — |
| 19 | | | 65 | 68 | 58 | .75 | .78 | 45 | 44 | W | W | — | — | — |

The quantity of Rain fallen in April, was 2 inches and 28-100ths.

